# *In vivo* test of *Vibrio alginolyticus* and *Vibrio harveyi* infection in the humpback grouper (*Cromileptes altivelis*) from East Java Indonesia

**DOI:** 10.14202/vetworld.2022.1269-1282

**Published:** 2022-05-23

**Authors:** Uun Yanuhar, Hendra Nurcahyo, Luluk Widiyanti, Nur Sakinah Junirahma, Nico Rahman Caesar, Sukoso Sukoso

**Affiliations:** 1 Department of Waters Resources Management, Faculty of Fisheries and Marine Science, Brawijaya University, Malang 65145, East Java, Indonesia; 2 Master Program of Aquaculture, Faculty of Fisheries and Marine Sciences, Brawijaya University, Malang, Veteran Street, Malang 65145, East Java, Indonesia; 3 Doctoral Program of Environmental Science, Postgraduate Program, Brawijaya University, Malang 65145, East Java, Indonesia; 4 Department of Fishery Product Technology, Faculty of Fisheries and Marine Sciences, Brawijaya University, Malang 65145, East Java, Indonesia

**Keywords:** crude protein, humpback grouper, *Vibrio alginolyticus*, *Vibrio harveyi*, western blot

## Abstract

**Background and Aim:**

The need for fish seeds resistant to bacterial and viral infections has encouraged studies on the molecular pathogenesis mechanism of *Vibrio* bacteria, such as *Vibrio alginolyticus* and *Vibrio harveyi*, regarding the receptor organs, protein adhesion mechanisms, and antibody responses of the humpback grouper. This study aims to confirm the characteristics of the specific proteins expressed in the receptor organ of the humpback grouper (*Cromileptes altivelis*) using the expression of *V. alginolyticus* and *V. harveyi* bacteria.

**Materials and Methods:**

The study was conducted by isolating crude protein and whole cells from both the *Vibrio* bacteria. In addition, serum and organ tissue were also isolated from fish samples. Then, hemagglutination and dot blot tests with polyacrylamide gel electrophoresis analysis were performed to determine the highest expression of receptor from the whole bacterial cells and crude protein from both healthy and infected (*V. alginolyticus* and *V. harveyi*) fishes. Scanning electron microscope results showed that *V. alginolyticus* and *V. harveyi* could express bundle-forming pili, which is involved in bacterial autoaggregation and the mediation of the initial attachment of bacteria to their host cells.

**Results:**

These results indicated that all the specific receptors for protein in fish organs recognized vibriosis antigens. The specificity test showed that the brain, eye, and kidney organs’ receptors provided a quality and quantity level of responses at 22.63, 53.95, and 43.15 kDa, respectively. The polyclonal anti-*V. alginolyticus* immunoglobulin M (IgM) antibodies were more cross-reactive than the anti-*V. harveyi* IgM. Hence, this shows that *V. alginolyticus* bacteria are more pathogenic than *V. harveyi*.

**Conclusion:**

In the future, the molecular characteristics of *V. alginolyticus* and *V. harveyi* antigens and the specific receptor organ proteins in the humpback grouper can be developed as the basis for constructing molecular peptide-based vaccine materials.

## Introduction

Grouper cultivation is a commodity with high economic value in several countries, especially in Asia. According to FAO [1], almost 155,000 tons of grouper fishes are produced and cultivated in China (Taiwan Province) and Indonesia, which is worth 630 million USD. Moreover, Indonesia has a strong grouper aquaculture industry, mostly in Situbondo, that contributes approximately 11% of the global production. Grouper fish production increased from 269,179 to 578,776 tons from 2012 to 2017 [2-4]. Monoclonal antibodies to detect *Vibrio harveyi* helped determine the concentration of the bacteria, 10^7^-10^8^ colony-forming units (CFU)/mL in pure culture, with a dot blot [5]. However, disease slows the growth of the fish. Almost 50% of lost production in aquaculture is caused by diseases, especially in developing countries. High stocking density and poor water quality lead to pathogenic levels of bacteria because they provide optimal conditions for parasite infestation and reproduction. In addition, infections spread during fish transport [6-8]. The inadequate preparation of disease-resistant seed is a major problem that has caused the declination of grouper cultivation, especially in Indonesia.

Furthermore, the *Vibrio* species have the potential to cause vibriosis within groupers [9]. They are the primary bacterial disease in the seed phase of marine culture or brackish fishes, such as the humpback grouper (*Cromileptes altivelis*). These *Vibrio* diseases cause approximately 100% mortality within 2 weeks. In addition, environmental effects can cause them to become malignant when the conditions are more supportive of bacterial rather than host survival [10]. Moreover, these *Vibrio* species are opportunistic pathogens that capitalize on previous infections and environmental stresses, including tidal changes or decreases in water quality [2,11,12]. *Vibrio alginolyticus* and *V. harveyi* are Gram-negative, motile, rod-shaped bacteria that are often found in marine and estuarine environments. These bacteria are closely related to severe disease outbreaks in most commercial marine fishes. The *V. alginolyticus* is reported in aquatic animals, including fishes, crustaceans, shellfish, and coral reefs. *V. alginolyticus* and *V. harveyi* infections occur in the cultivation of Cobia, Rachycentron canadum, Asian Seabass, and Lates calcarifer [13-16]. *V. harveyi* causes severe illness and death in many aquaculture species, resulting in substantial global economic losses. The marine species infected with these bacteria include penaeid shrimp, rainbow trout, Atlantic salmon, olive flounder, black rockfish, sea bream, and turbot [2,12,17,18].

Poor water environmental conditions can easily cause stress for fish because they also experience a decrease in their bodies’ defense system against pathogens [6]. However, when the water environment is ideal, weak (sick) fishes still have the potential to be infected with bacteria. These bacteria are either transmitted through water or direct contact, resulting in a quick distribution to fish grown at a high density. *V. alginolyticus* causes exophthalmia, wounds, septicemia, corneal cloudiness, and death of fish and shrimp [19]. In addition, these bacteria have an array of mechanisms for developing their colonization. These combined strategies help modify secretion pathways to increase the surface molecules necessary for colonization or convey effector molecules to other bacteria. *Vibrio* bacteria’s infection mechanism produces adhesive molecules, which function as an attachment between bacterial protein substance and their cognate receptors during the infection of host cells. The attachment of bacteria to host cells, which includes several different bonding mechanisms, requires adhesin and host receptors. Complement receptors and adhesin molecules form bonds in the contact area between the two cells. Adhesions under physiological conditions become irreversible. In addition, host cell surface receptors rearrange the cytoskeleton, resulting in changes in the regulation of the cell’s protein (proteomic function). This indicates that the expression of adhesive protein occurs within the host cell, and infected grouper fish form antibodies within their body.

Pathogens selectively colonize the surface of the small intestine while growing and attacking the tissue with virulence factors, which causes tissue damage and leads to disease [20]. Infected fish show symptoms of anorexia, unresponsiveness, low vitality, and weakness during the early stages of the disease. As the infection worsens, more severe symptoms such as crusting, epidermal bleeding, and canker sores on the skin and mouth of the fish appear. In the most severe cases of infection, the tail shaft is ulcerated into the muscles. Fish with vibriosis often have swollen spleens, loose stomachs, and colitis [12]. The bacteria can trigger activation of the immune system. Antigens entering the host cell will be presented by Class I (major histocompatibility complex) and captured by receptors on T cells (2). T cells secrete cytokines interleukin (IL)-2, IL-4, and IL-6 to differentiate and induce B cell proliferation. B cells will differentiate into memory cells and plasma cells. Furthermore, plasma cells will synthesize antibodies that will specifically bind their antigens, preventing the movement of antigens and facilitating the process of phagocytosis [21]. Thus, the adhesion protein should be reviewed.

Hence, vaccination is appropriate preventive strategy because antibiotics result in the accumulation of resistance. In addition, several resistant strains of *V. harveyi* and *V. alginolyticus* have caused severe economic setbacks in Asia and Latin America [22]. The molecular pathogenesis of *V. alginolyticus* and *V. harveyi*, based on the receptor organs, adhesive protein mechanisms, and immunoglobulin M (IgM) antibody production in the humpback grouper, has not been widely detected. The previous studies [6,17,23] have shown that vaccines consisting of immunogenic fractions induce higher protection than attenuated whole-cell bacteria in fish. The characteristics of the specific receptor protein in the humpback grouper are used to make vaccines. These vaccines recognize several *Vibrio* bacteria adhesive proteins to support the production of superior seeds in humpback grouper cultivation.

This study aimed to confirm the characteristics of specific proteins expressed in the receptor organ of the humpback grouper (*C. altivelis*) infected by *V. alginolyticus* and *V. harveyi*.

## Materials and Methods

### Ethical approval

The Research Ethics Commission, University of Brawijaya, has carefully studied the research design and provided ethical approval (118-KEP-UB-2021).

### Sampling and maintenance of humpback grouper

The samples of humpback groupers were obtained from the Brackish Water Hatchery Centre (BPBAP) in Situbondo. The study used 12 aquarium units with a capacity of 60L. Fifty fish samples with sizes of ±9–10 cm were taken in 5 aquariums containing ten fish each. Another 2 aquarium units were used for infection treatment, and 5 aquarium units were used for maintenance aquariums after infection. The maintenance aquarium was acclimatized before the hatchery of fish by sterilization with KCl, and then it was dried for 24 h. Then, it was soaked in freshwater for 2 days. Next, the freshwater was discarded and replaced by seawater with a 30-33 ppt salinity level. The aquarium was sterilized with 100 mg/L chlorine (CaCOl_2_) and left for 3 days. Aeration was installed to evaporate the remaining chlorine. After 3 days, the seawater was neutralized using sodium thiosulfate (Na_2_S_2_O_3_) with a concentration of 100 ppt. The aquarium was equipped with aeration, a heater, and a closed recirculation system to maintain oxygen and temperature stability.

### Identification of *V. alginolyticus* and *V. harveyi* bacteria

The isolates of the *V. alginolyticus* and *V. harveyi* bacteria, which were obtained from BPBAP laboratory, were routinely grown in a Tryptic Soy Agar (TSA; Difco, USA) or Tryptic Soy Broth; Difco, with 0.5% NaCl at 25°C. The cultured stock was maintained, as a suspension with 25% (v/v) glycerol, at −80°C. Bacterial identification was based on colony morphology, gram characteristics, motility, and cells. In addition, biochemical testing was conducted by analyzing a microbat system. *V. alginolyticus* and *V. harveyi* were tested using standard biochemical diagnostic kits (Becton Dickinson and Company, USA) [24,25]. Bacteria were identified through 16S rRNA sequencing, and the total genomic DNA was extracted from the samples by the EZ-10 Spin Column Bacteria Genomic DNA Miniprep Kit (Bio Basic, Canada) [26]. Morphological observations of *V. alginolyticus* and *V. harveyi* were made on isolated and pure isolates. Furthermore, the form and edges shape of the colonies that grew on TCBSA culture media at room temperature (30°C-32°C) for 48 h were noted. These bacteria were inoculated using a loop needle in nutrient agar and TCBSA media (Merck, Germany). The incubation was carried out at 30°C-32°C for 24 h to calculate the CFU. The remaining bacteria were collected by centrifugation at 300× *g* at 4°C. They were then washed 3 times in 1× binder buffer (50 mM Tris-HCl (Merck), 100 mM NaCl (Merck), 5 mM KCl (Merck), and 1 mM MgCl_2_ (Merck), pH 7.4) [27].

### Isolation of whole-cell and crude protein of *V. alginolyticus* and *V. harveyi*

The procedure for isolating whole cells and crude protein from *V. alginolyticus* and *V. harveyi* is adapted from Bunpa *et al*. [28], with several modifications (The difference lies in the speed of centrifugation (4000 × g for 30 min) and the number of bacteria (2.53×107 CFU/g), then homogenize the pellet with the addition of 0.05% NOG before being centrifuged again). The bacteria in 500 mL of BHI media were centrifuged to obtain whole cells and crude protein. The MSE centrifuge was run at 3578 × g and for 10 min. Next, 50 mL of each bacterial growth (*V. harveyi* and *V. alginolyticus*) were placed in 50 mL of eppI in Eppendorf falcons (Onemed, Indonesia). The cell pellets were resuspended in lysis buffer (7 M urea, 2 M thiourea (Mettler Toledo™, Switzerland), 4% 3-[(3-cholamidopropyl)dimethylammonio]-1-propanesulfonate (CHAPS, Thermo Fisher Scientific, USA), 2% pharmalite (GE Healthcare, UK) pH 4-7, and 40 mM dithiothreitol [DTT] Thermo Fisher Scientific), and incubated at 4°C for 1 day. Then, the crude cell homogenate was sonicated and centrifuged at 14,000× *g* for 5 min, at 4°C. Supernatants were collected and stored at −70°C. The cell pellets were homogenized by grinding in a mortar to lyse the bacterial cell walls; 0.05% N-Octyl-Glucopiranoside (NOG, MilliporeSigma, Germany) was added before centrifugation at 4°C for 20 min at 8117 × g. The resulting pellets were whole cells and the supernatant was crude protein.

### *V. alginolyticus* and *V. harveyi* bacterial infection against humpback grouper

The two *V. alginolyticus* and *V. harveyi* infection aquariums were 30×30×20 cm, had a volume of 16 L, and were prepared with aeration. The infected fish were 10-15 cm in length, and there was a maximum of two fish per aquarium. A challenge test for the infection of *V. alginolyticus* and *V. harveyi* within humpback groupers was conducted to obtain the infected fish. *V. alginolyticus* and *V. harveyi* were obtained from a bacterial crop cultured previously. The bacterial density was calculated using an indirect method based on the number of colonies. After acclimatization, fish were infected with approximately 1.25×10^6^ CFU/mL of *V. alginolyticus* and *V. harveyi* bacteria placed in the aquarium using the immersion method. Fish were observed and analyzed 5 days after infection. The placement of the aquarium is in a closed room. The water quality condition of the aquarium was maintained with aeration without killing the *V. alginolyticus* and *V. harveyi* bacteria. Fishes were still given food intake and observed their changes in behavior. After five days, the fishes infected with *V. alginolyticus* and *V. harveyi* bacteria were transferred to another aquarium.

### Protein isolation of the humpback grouper receptor organ

Organ receptor protein was isolated from healthy and infected humpback groupers. The samples infected with *Vibrio* were stored in liquid nitrogen. Yanuhar’s method was used to isolate the brains, eyes, and kidneys from the groupers in a laminar flow hood with sterile scalpels [29]. These organs were homogenized with a sterile mortar, and nerve cells were isolated by adding a buffer extract at a ratio of 2 mL:1 mg. This homogenate was centrifuged at 1677 × g for 1 h to separate the debris from the nerve cells in the brains. The morphology of the degraded nerve cells was examined, and the receptor protein was isolated. Next, samples were further centrifuged at 15,093 × g for 3-5 h to obtain *Vibrio* protein. The supernatant was separated from the pellets, showing that *Vibrio* crude protein was obtained. Then, it was packaged and stored in a sterile Eppendorf in a −80°C freezer.

### Isolation of fish blood serum of humpback grouper

On the 10^th^ day after infection, three fish per tank were randomly selected for a blood sample. The fish were starved for 24 h before sampling and anesthetized in freshwater containing 406 mg/L 2-phenoxyethanol (ethylene glycol monophenyl ether, Sigma, Germany). Peripheral blood was obtained from the caudal vein by a 23-gauge needle with a 1 mL nonheparinized syringe. This blood was allowed to freeze at 4°C for 4 h. Then, the blood clots were centrifuged out at 10,000× *g* for 10 min and stored at −20°C for hemagglutination analysis [30].

### Hemagglutination test for *V. alginolyticus* and *V. harveyi* bacteria

According to the previous research [31,32], the hemagglutination test was modified. The erythrocytes from healthy fish were washed with phosphate-buffered saline (PBS, 7.4), shaken gently to be homogeneous, and centrifuged at 822 × g for 10 min twice. A 50 μL erythrocyte dilution was added into a tube until 10 mL were homogenized and ready to be used for the HA test. The sample was further diluted with ½ concentration in micro plan V for each well, with a volume of 50 μL. Furthermore, each well was added with erythrocytes at a similar volume concentration of 0.5%. It was added to 50 µL of PBS in wells 1-10 and 12, with 11 observed to be empty. The diluted sample was added to 50 µL bacterial antigen (whole-cell and crude bacterial protein in different wells) in wells 1 and 2. It was also added to 50 µL PBS in wells 1 and 2. Starting from well 2, the diluted sample was pipetted with 50 µL into well 3, and this was repeated until well 10. Erythrocytes were added to wells 1-10 and 12, and the resulting reactions were awaited. The titer value was determined by the presence of erythrocytes agglutination at the lowest dilution. The samples tested were the whole cells and crude protein of *V. alginolyticus* and *V. harveyi*, respectively. The erythrocyte cells used were from the humpback grouper (*C. altivelis*). Positive reactions occurred when the whole cells and bacterial proteins agglutinated erythrocytes, indicated by an absence of an erythrocyte dot on the well. By contrast, erythrocytes marked by a dot on the well bottom indicated a negative reaction.

### Anti-adhesin IgM grouper specificity test for *V. alginolyticus* and *V. harveyi* through dot blot technique

The specificity of the grouper IgM antibody was tested using the dot blot method with *V. alginolyticus* and *V. harveyi* antigens and the specific receptor organ of the fish [33]. First, nitrocellulose (NC) was soaked in PBS for 30 min in the dot blotting chamber. The NC was inserted into healthy fish receptor organs in 5% skimmed 50 µL PBS (1:10) and incubated at 30-32°C for 1 h. The crude protein *V. alginolyticus* and *V. harveyi* antigen were added with 5% skimmed 50 µL PBS (1:10) and incubated overnight at 30-32°C. Then, the incubation results were blocked with 5% skimmed PBS for 1 h and washed with PBS Tween 0.05% (5×5 min). Then, this was added to primary antibodies from *Vibrio*-infected fish serum (1:50) with 5% skimmed PBS at 50 µL and incubated overnight at 30-32°C. The results were washed with Tween 0.05% (5×5 min), and the secondary antibody was added (antigrouper) (1:50) in 5% skimmed PBS at 50 µL. Fifty microliters of the chromogen substrate, NBT, were added and incubated at 30-32°C for 2 h. The reaction was stopped by immersion in 50 µL of distilled water. The results revealed color gradations that could be analyzed using the Corel Draw Software to determine average values [34]. To determine the specificity level of the organ receptor protein against the antibodies produced by infected humpback grouper, the dot blot test was done without adding *Vibrio* bacterial crude protein.

### Anti-adhesin IgM grouper antibody specificity test for *V. alginolyticus* and *V. harveyi* through western blot technique

The western blot technique used was based on Hnasko’s method [35]. Sodium dodecyl sulfate-polyacrylamide gel electrophoresis (SDS-PAGE) was used to separate the proteins. Then, one gel was transferred to NC paper over a semidry device. This gel was stained with 2% poncho dye, which contained approximately 3% TSA to determine whether the sample protein had moved to the NC paper. In addition, the gel was marked to determine the molecular weight. The NC paper was cut according to the rows of the wells. Antigrouper IgM with a secondary antibody was added at a concentration of 1/1000 in TBE with a pH of 7.4 and BSA 1% (Thermo Fisher Scientific) and protected against light. Shaking was done for 2 h, and it was washed twice for 5 min, by 0.05% TBE pH 7.4 tween 20. Then, β-cip tablets dissolved in 10 mL H_2_O were used as a color agent. This solution was poured into the NC paper and observed for the occurrence of red color. When the reaction was complete, it was rinsed with H_2_O and dried with filter paper.

### Protein fractionation with SDS-PAGE

Using SDS-PAGE, hydrolyzed samples from various treatments were analyzed [36,37]. The samples (containing 40 µg protein) were mixed 1:1 with loading buffers that contained 0.125 M Tris-HCl, 4% SDS, 20% v/v glycerol, 0.2 M DTT, and 0.02% bromophenol blue (Merck) at a pH of 6.8. This diluted sample was preheated for 5 min in a boiling water bath at 100°C. The analysis was performed in a vertical electrophoresis unit (SE 260, Hoefer, San Francisco, CA) with 1.5 mm (17%) polyacrylamide gel slabs to separate the protein, at a constant current of 30 mA per gel. The separated protein bands were stained with a solution containing 7% acetic acid(Merck), 0.5% Coomassie Brilliant Blue (Thermo Fisher Scientific) R-250, and 40% methanol (Germany). This excess stain was removed with a solution containing 40% methanol and 7% acetic acid, with the gel was recorded with an electronic scanner (Umax Power Look 2100, UMAX Technologies, Fremont, CA, USA).

## Protein dialysis of SDS-PAGE results

Protein dialysis was used to analyze the results of the SDS-PAGE. The gel pieces containing *V. alginolyticus* and *V. harveyi* protein bands were purified by the electroelution method to remove protein through horizontal electrophoresis (Bio-Rad, USA), which was done by inserting a piece of proteinous gel into the cellophane membrane (Merck, USA). This membrane already contained a buffer of pH 8.3 electrodes, and it was kept at 25 V at room temperature for 150 min until the gel pieces cleared. The solution in the cellophane membrane was transferred to a new location to proceed to the dialysis stage. The dialysis was accomplished by inserting protein pieces into a new cellophane membrane for 24 h, which contained a PBS solution pH 7.4 at 4°C. Electrophoresis was conducted at 120 V or 400 mA for 120 min. The liquid in the cellophane bag was placed in a microtube and precipitated by incubating in acetone solution (1:1 v/v) overnight at 4°C. The protein and acetone mixture was centrifuged at 9660 × g for 20 min at 4°C. The protein formed pellets were dried and then dissolved in 100 µL of 0.5 M tris-HCl at pH 8.6. Nanodrop Spectrophotometry ND 1000 measured protein concentration with an absorbance of 1 at 280 nm. Successful purification was confirmed using the SDS-PAGE method [38,39].

### Clinical test of the effectiveness of the bacterial proteins of *V. alginolyticus* and *V. harveyi* on humpback grouper

The provision of proteins purified by dialysis was used as an oral injection vaccine against normal humpback grouper. The fish were provided with feed treatment under normal conditions (trash fish twice a day). Vaccines were administered on the 1^st^ day of treatment and then boosted. These treatments include (a) healthy grouper, (b) grouper with *V. alginolyticus* infection, (c) grouper with *V. harveyi* infection, (d) grouper with immunogenic protein of *V. alginolyticus*, and (e) grouper with immunogenic protein of *V. harveyi*. The serum was obtained and isolated based on the production of antibodies for 13 days. Immunochemical techniques subsequently confirmed this through the dot and western blots methods. Visual observations of normal fish organs were made using a scanning electron microscope (SEM) (Hitachi SU70, Japan).

### SEM

Observations using an SEM were based on the study of Li *et al*. [40]. Each bacterial *V. alginolyticus* and *V. harveyi* culture in LB medium to mid-log phase were harvested by centrifugation at 1000× *g* for 10 min. The cell pellets were washed twice with 10 mmoL/L of PBS and resuspended to an OD600 of 0.2. The cell suspension was incubated at 27-30°C for 30 min with different peptides at a concentration of 1 MIC. The suspension was centrifuged and washed 3 times at 5000× *g* for 5 min, with PBS as control. The bacterial pellets were fixed in 500 mL of 2.5% (v/v) glutaraldehyde in PBS at 4°C overnight. The bacteria were washed twice with PBS and dehydrated through a stratified ethanol sequence (50%, 70%, 90%, and 100%) for 15 min each. The samples were transferred to a 1:1 (v/v) mixture of ethanol and tertiary butanol and a pure compound (tertiary butanol) for 20 min each. After lyophilization and gold coating, the specimens were observed using an SEM.

## Results

### Observation of clinical symptoms

**Figure-1:**
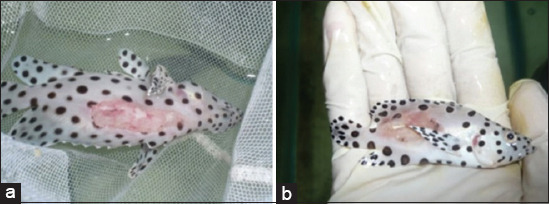
Clinical symptoms of *V. alginolyticus* and *V. harveyi* bacterial infection, wounds on the body and gills of the fish are reddish, (a) red sores that appear in fish infected with *V. alginolyticus*, (b) red sores that appear in fish infected with *V. harveyi. V. harveyi*=*Vibrio* *harveyi*, *V. alginolyticus*=*Vibrio* *alginolyticus*.

The symptoms of *Vibrio* infection appeared swirling, such as loss of balance and partial settling on the pond’s bottom. The symptoms of a vibriosis attack presented as a gaping wound that looked like a burn, with reddish tissue around the injury (Figure-1). There were also other symptoms, such as the collection of mucus on the infected wound, thinning of fins, and bulging stomachs and eyes. Furthermore, the physiological symptoms examined in infected fishes were reduced appetite, behavioral changes, sluggish movements, and weakness. The behavioral changes occurred in fish with *V. alginolyticus* and *V. harveyi* within 3-12 h after infection.

### Cultivation of *V. alginolyticus* and *V. harveyi*

**Figure-2:**
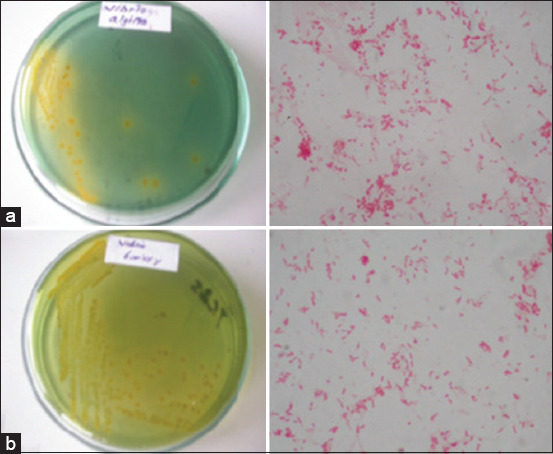
Results of TCBS agar culture and staining bacterial observations. (a) *Vibrio alginolyticus* and (b) *Vibrio harveyi*.

The two representative strains of *Vibrio* bacteria isolated from different groupers were subjected to further analysis. Both isolates were Gram-negative and morphologically homogeneous in rod formation; the observation results from the bacterial cultures of *V. alginolyticus* and *V. harveyi* (Figure-2).

Furthermore, both bacteria (*V. alginolyticus* and *V. harveyi*) were isolated from the brackish waters of floating net cages in grouper culture, which indicated vibriosis based on the appearance of symptoms. They were cultured in physiological Na conditions of 0.5% on BHI media. These bacteria were selective on TCBS agar media, which was indicated by the colony shape of each bacterial species.

### Infected-organ observation using the SEM

**Figure-3:**
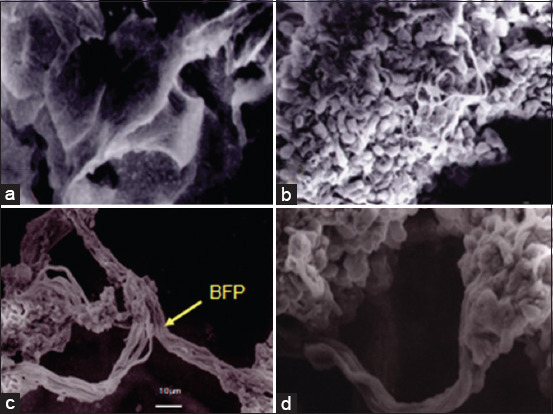
Observation of organs and bacteria (examination with a Scanning Electron Microscope). (a) Epithelial cell receptors for fish organs, (b) *Vibrio alginolyticus* bacterial infection, (c and d) bundle-forming pili during the infection process in organs.

The SEM showed that t *V. alginolyticus* and *V. harveyi* cells were both Gram-negative short rods, as shown in Figure-3. The grouper receptors tested and found the primary receptors in the eye and kidney organs indicated by specific protein expressions, which had recognized *V. alginolyticus* and *V. harveyi* antigens.

### Analysis for the whole-cell protein of Vibrio spp. as well as the crude protein of *V. alginolyticus* and *V. harveyi*

**Figure-4:**
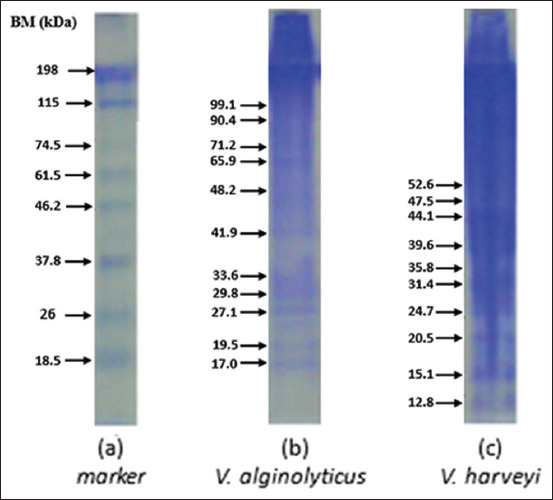
Sodium dodecyl sulfate-polyacrylamide gel electrophoresis results for the whole cell of *Vibrio* spp. using 0.05% NOG.

**Figure-5:**
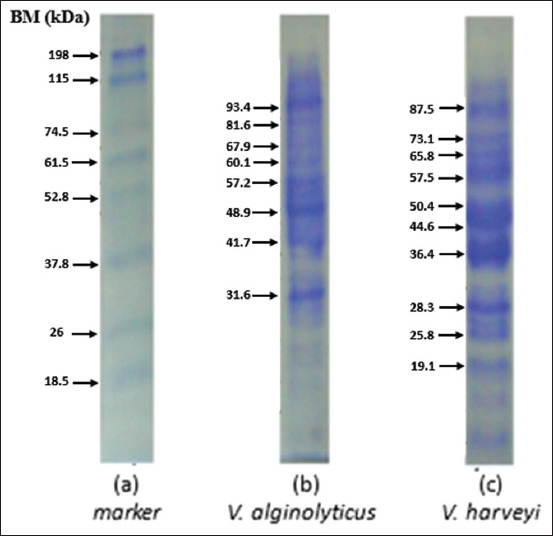
Sodium dodecyl sulfate-polyacrylamide gel electrophoresis results for the crude protein of *Vibrio* spp. using 0.05% NOG.

Figures-4 and 5 showed that the whole-cell and crude proteins in *V. alginolyticus* and *V. harveyi* bacteria isolated with 0.05% NOG differed in molecular protein weight. The results of the whole-cell analysis of *V. alginolyticus* were 31.6, 41.7, 48.9, and 57.2 kDa. *V. harveyi* has molecular weights of 28.3, 36.4, 44.6, 50.4, and 57.5 kDa. Meanwhile, the crude protein molecular weight of *V. alginolyticus* was 32.5, 42.8, 55.2, and 67.9 kDa. The crude protein molecular weight of *V. harveyi was* 36.4, 47, 63.4, and 83.5 kDa. The high molecular weight protein may be part of the siderophore system.

### Analysis for immunogenic adhesin protein-specific response of *Vibrio* spp., which recognizes receptor organs

**Figure-6:**
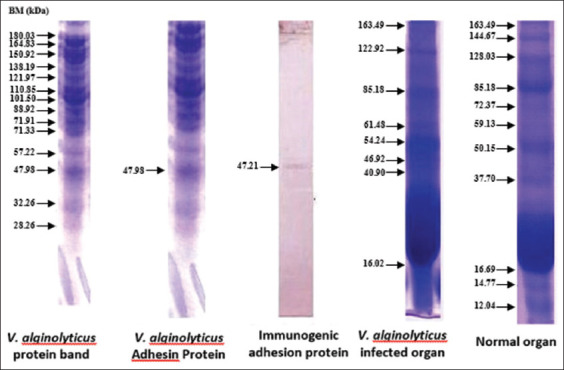
Immunogenic protein response of *Vibrio alginolyticus* in the eye organs.

**Figure-7:**
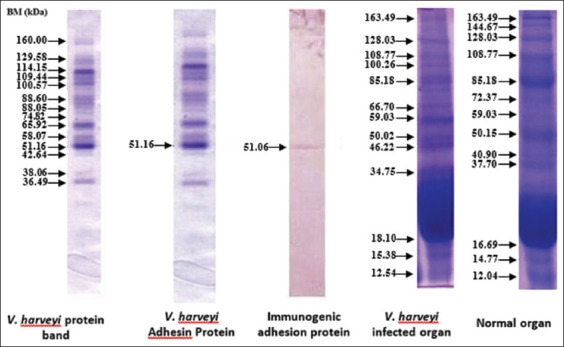
Immunogenic protein response of *Vibrio harveyi* in the eye organs.

The results of *V. alginolyticus* immunogenic protein-specific response to the eye receptor organ in Figure-6 showed the difference in molecular protein weight, which was further analyzed to determine the expression that played a role in infecting other organs. After purification, this specific adhesion and immunogenic proteins of *V. alginolyticus* appeared at 47.98 and 47.21 kDa, respectively. Figure-7 showed that the adhesion and immunogenic proteins of *V. harveyi* appeared at 51.16 and 51.06 kDa, respectively.

### Hemagglutination test for *V. alginolyticus* and *V. harveyi*

Table-1 presents the results of the receptor specificity test with the hemagglutinin.

**Table 1:** The HA test results of the grouper fish receptor which has cross-reaction with the antigen protein from the whole-cell of *Vibrio* spp.

* Well	Dilution dose										Control

	1/2	1/4	1/8	1/16	1/32	1/64	1/128	1/256	1/512	1/1024	
A	+	+	+	+	+	+	+	+	+	+	−
B	+	+	−	−	−	−	−	−	−	−	−
C	+	−	−	−	−	−	−	−	−	−	−
D	+	+	+	−	−	−	−	−	−	−	−

* A=Whole-cell of *V. alginolyticus*. B=Protein matrix of *V. alginolyticus*. C=Whole-cell of *V. harveyi*, D=Protein matrix of *V. harveyi. V. harveyi*=*Vibrio harveyi, V. alginolyticus*=*Vibrio alginolyticus*

Table-1 shows that the whole protein of *V. alginolyticus* had the highest hemagglutinin dose until 1/1024 dilution. Figures-6 and 7 present the identification results for the immunogenic adhesion protein response of *V. harveyi* and *V. alginolyticus*. Table-2 shows the results of the hemagglutinin test for the treatment of *V. alginolyticus* immunogenic protein. Hemagglutinin was recognized in the whole protein of *V. alginolyticus* and was provided by a positive value until the 1/32 titer dilution. Meanwhile, *V. alginolyticus* (47.9 kDa) showed that 1/64 titer still provided a positive dilution value. Table-3 shows the protein dilution titer of *V. harveyi* bacteria. The whole protein of *V. harveyi* was supplied by a positive value until the 1/64 protein dilution. Then, the protein matrix from *V. harveyi* (51.16 kDa) showed approximately 1/16 titer. The results indicated that both *V. alginolyticus* and *V. harveyi* bacteria had adhesin proteins, namely, hemagglutinin pili, with molecular weights of 47.98 and 51.16 kDa, respectively.

**Table 2:** The HA test results of the observations on the dilution of *V. alginolyticus*.

* Well	Dilution dose										Control

	1/2	1/4	1/8	1/16	1/32	1/64	1/128	1/256	1/512	1/1024	
A	−	−	−	−	+	+	+	+	+	+	+
B	+	+	+	+	+	+	+	+	+	+	+
C	+	+	+	+	+	+	+	+	+	+	+

* A=Whole-cell of *V. alginolyticus* (HA+4). B=Protein matrix of *V. alginolyticus* 101 kDa (HA+1). C=Protein matrix of *V. alginolyticus* 47.9 kDa (HA+1). *V. harveyi=Vibrio harveyi, V. alginolyticus=Vibrio alginolyticus*

**Table 3:** The HA test results of the observations on the dilution of *V. harveyi.*

* Well	Dilution dose										Control

	1/2	1/4	1/8	1/16	1/32	1/64	1/128	1/256	1/512	1/1024	
A	−	−	−	+	+	+	+	+	+	+	+
B	−	+	+	+	+	+	+	+	+	+	+
C	−	+	+	+	+	+	+	+	+	+	+
D	−	−	+	+	+	+	+	+	+	+	+
E	−	−	−	+	+	+	+	+	+	+	+

* A=Whole-cell of *V. harveyi*, B=Protein matrix of *V. harveyi* 114.16 kDa, C=Protein matrix of *V. harveyi* 65.92 kDa. D=Protein matrix of *V. harveyi* 51.16 kDa. E=Protein matrix of *V. harveyi* 36.49 kDa. *V. harveyi=Vibrio harveyi, V. alginolyticus=Vibrio alginolyticus*

### Analysis of brain, eye, and kidney receptor organ proteins in humpback grouper specific to the antigen

**Figure-8:**
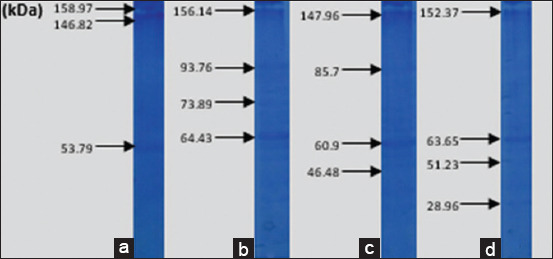
The sodium dodecyl sulfate-polyacrylamide gel electrophoresis results of organ receptor protein for healthy grouper using NOG 0.05%. (a) Markers, (b) brain, (c) eyes, and (d) kidneys.

**Figure-9:**
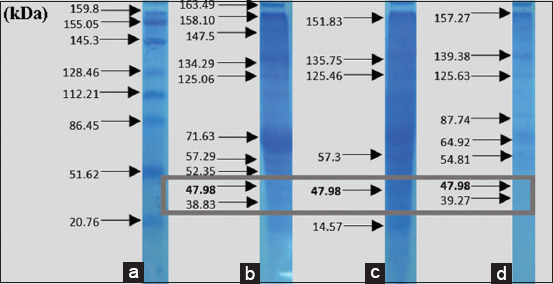
The sodium dodecyl sulfate-polyacrylamide gel electrophoresis results of organ receptor protein for grouper infected with *Vibrio alginolyticus* of 47.98 kDa using 0.05% NOG. (a) Markers, (b) brain, (c) eyes, and (d) kidneys.

**Figure-10:**
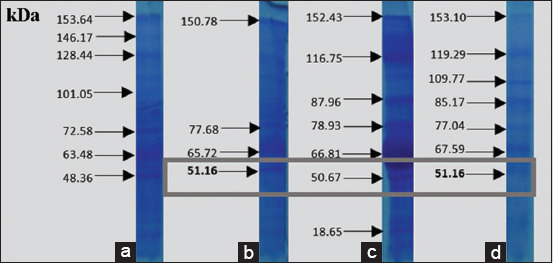
The sodium dodecyl sulfate-polyacrylamide gel electrophoresis results of organ receptor protein for grouper infected with *V. harveyi* of 51.16 *kDa* using 0.05% NOG. (a) Markers, (b) brain, (c) eyes, and (d) kidneys.

**Figure-11:**
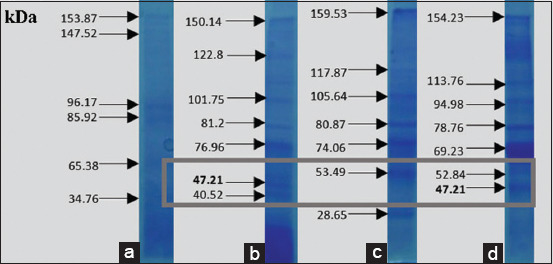
The sodium dodecyl sulfate-polyacrylamide gel electrophoresis results of organ receptor protein for grouper with *Vibrio* *alginolyticus* immunogenic protein of 47.21 kDa using 0.05% NOG. (a) Markers, (b) brain, (c) eyes, and (d) kidneys.

**Figure-12:**
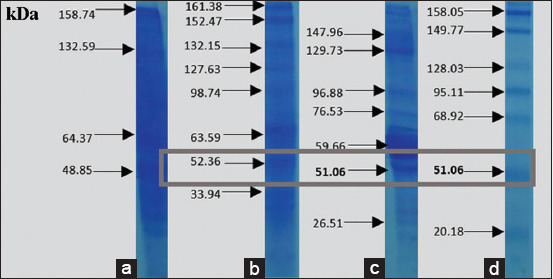
The sodium dodecyl sulfate-polyacrylamide gel electrophoresis results of organ receptor protein for grouper with *Vibrio harveyi* immunogenic protein of 51.06 kDa using 0.05% NOG. (a) Markers, (b) brain, (c) eyes, and (d) kidneys.

Figures-8-12 show the SDS-PAGE electrophoresis results for the different organ profiles of the humpback grouper.

The SDS-PAGE using 0.05% NOG showed the presence of protein bands with different thicknesses. The thickness and thinness of the protein bands seen on the polyacrylamide gel illustrate the amount of protein contained in the molecular weight. Based on Figure-8, the healthy fish had a brain, eye, and kidney organs molecular weight of 64.43, 60.9, and 63.65 kDa, respectively. Meanwhile, the bands on *V. alginolyticus* (Figure-9) indicated the molecular weights of 71.63, 57.3, and 64.92 kDa for the brain, eye, and kidney proteins, respectively. Furthermore, the molecular weight observed in Figure-10 indicated the specific role of the receptor in antigen recognition, that is, 65.72, 66.81, and 67.59 kDa for the brain, eye, and kidney, respectively. The treatment of *V. alginolyticus* immunogenic protein (Figure-11) on the brain, eye, and kidney receptor organs was 76.96, 74.04, and 69.23 kDa, respectively. The treatment of *V. harveyi* immunogenic protein (Figure-12) on the brain, eye, and kidney receptor organs was at 63.59, 59.66, and 95.11 kDa, respectively.

### Specificity test of antibody IgM grouper-anti-adhesin *V. alginolyticus* and *V. harveyi* through dot blot technique

The results were measured in quantity, indicating that *V. alginolyticus* had a significant pathogenic infection incidence (Table-4).

**Table 4:** Clinical test results on the quantification of grouper-specific receptor examinations (eyes, brain, and kidney), using a dot blot and analysis using the Corel 12 software.

Receptor	*V. alginolyticus* matrix		*V. harveyi* matrix	
	
	Ig. *V. alginolyticus*	Ig *V. harveyi*	Ig. *V. alginolyticus*	Ig *V. harveyi*
Eyes	69.46	84.8	87.6	77.35
Brain	88.92	64.95	80.54	82.75
Kidney	55.8	51.5	60	87

Quantification color value: Black=0; White=255

**Figure-13:**
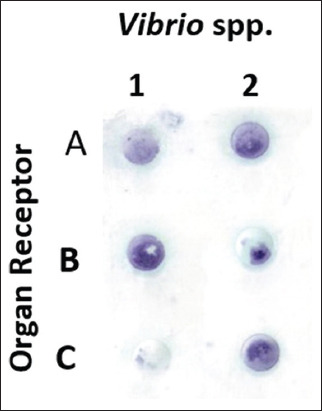
Specifications of the brain, eye, and kidney organ protein receptor units against the *Vibrio* spp. with the dot blot technique. (Organ receptors, A: Eye; B: Kidney; C: Brain; 1: *Vibrio alginolyticus*; 2: *Vibrio* *harveyi*).

The cross-reaction of antigens with antibodies was consecutively *V. alginolyticus*, with an average quantification of the color value of 69.24 (Table-4) using the Corel Draw 12 software. This was accompanied by *V. harveyi*, with an average quantification color value of 79.2. Based on the confirmation tests, the brain, eye, and kidney organs had receptor specificity, which recognized the *Vibrio* spp. antigen, as shown by the dot blot examination and quantification results in Figure-13 and Table-5.

**Table 5:** The results of the quantification test for the specificity of specific receptors against the antibodies to *Vibrio* spp.

Receptors	Ig. *V. alginolyticus*	Ig. *V. harveyi*
Eye	76.558	106.454
Kidney	124.24	60.352
Brain	30.718	98.709

Description: Black: 0; White: 255

**Figure-14:**
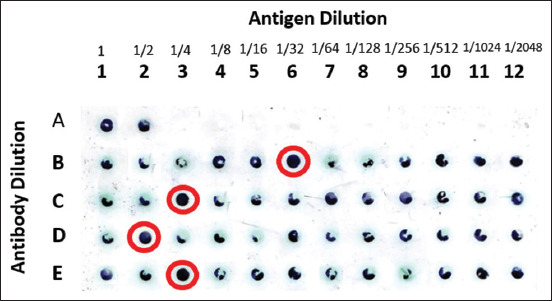
Clinical test results on receptor specificity testing using the dot blot technique against all *Vibrio* spp. protein matrix’s and anti-*Vibrio* spp. polyclonal antibodies; Columns 1-12 antigen dilution, Rows A-E antibody from fish serum ((a) positive control, (b) infected fish with *V. alginolyticus* of 47.98 kDa, (c) infected fish with *V. harveyi* of 51.16 kDa, (d) fish with *V. alginolyticus* immunogenic protein of 47.21 kDa, (e) fish with *V. harveyi* immunogenic protein of 51.06 kDa). *V. harveyi*=*Vibrio* *harveyi*, *V. alginolyticus*=*Vibrio alginolyticus*.

The dot blot quantification results showed that the kidney had a protein receptor with a specificity value of 124.24 for *V. alginolyticus* infection. The eye had a protein receptor with the highest specificity value of 106.454 for *V. harveyi* infection. The kidneys obtained the average value of the highest specificity. Therefore, the eye, brain, and kidney have specific receptors for introducing antigens against *V. algnolyticus* and *V. harveyi*. Figure-14 shows the results of the antigen-antibody reactions of receptor specificity between the *Vibrio* spp. protein matrix and anti-*Vibrio* spp. polyclonal antibody, using the dot blot method to determine the dilution titer with the strong response. The semiquantitative calculation of the antigen-antibody reaction was carried out with Corel Draw 12 software. The first two upper stripes were a positive control of post immune antibodies, which cross-reacted with antigens. The antibody dilution titer had a strong reaction at D2 (fish with *V. alginolyticus* immunogenic protein of 47.21 kDa) with an antigen titer dilution of 1/2. Furthermore, treatments C and E showed a reaction at titer 1/4. In treatment B, the reaction was at a titer of 1/32.

### Anti-adhesin IgM grouper antibody specificity test for *V. alginolyticus* and *V. harveyi* through Western blot technique

**Figure-15:**
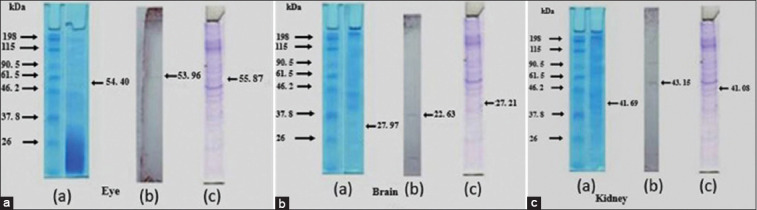
The results of the specificity test for the brain, eye, and kidney protein receptors exposed to anti-*Vibrio* spp. with western blotting technique, (a) whole-cell, (b) the specificity of immunoglobulin M antibodies in humpback grouper, and (c) marker.

Figure-15 shows the Western blotting test on the brain, eye, and kidney receptors against IgM anti-*Vibrio* spp. The results of the specificity test for matrix protein *Vibrio* spp. antigens in the fishes showed that the receptors in the brain, eye, and kidney organs provided a quality and quantity level of responses, at 22.63, 53.95, and 43.15 kDa, respectively.

## Discussion

The bacteria *Vibrio* spp. is one of the primary pathogenic organisms causing high mortality in the brackish aquaculture industry. The infections caused by bacteria belonging to the genus *Vibrio* are called vibriosis [41]. *Vibrio* spp. invades fish through oral ingestion, skin lesions, and gill surfaces. The typical clinical signs of vibriosis in septicemia and necropsy used in this study are in accordance with the investigations of Mohamad *et al*. [2], Xie *et al*. [41], Ina-Salwany *et al*. [42]. Figure-1 shows the most visible characteristic that wounds the body of fish. Skin lesions appear as the body’s natural reaction against pathogen infections such as bacteria.

Bacteria in chronic wounds can develop biofilms that contribute to the delayed healing process. Biofilms consist of sessile communities of several bacterial species covered by a protective carbohydrate-rich polymer matrix resistant to antimicrobial and immune cell penetration [43]. The *Vibrio* was easy to attack when the host organism had decreased immunity or physical stress. This infection often occurs in intensive cultivation due to poor environmental conditions. Both *V. alginolyticus* and *V. harveyi* infections led to higher levels of specific antibodies against both pathogens due to the local inflammatory effect described by the observations. Antibodies are specific humoral defenses to inhibit bacterial adhesion or non-phagocytic host cell invasion and neutralize bacterial toxins. Antibodies can activate complement through the classical pathway to lyse bacterial cells, activate inflammation, enter phagocytes, and further enhance phagocytosis. Macrophages and granulocytes are phagocytic mobile cells found in blood and secondary lymphoid tissues, usually found in acute inflammation cases, which is a cellular response to microbial invasion and tissue injury resulting in local accumulation of leukocytes and mucus fluid.

*V. harveyi* is a Gram-negative bacteria (Figure-2) that live in the ocean in tropical climates. Also, *Vibrio* species were found to emit light or bioluminescence [44]. According to Heenatigala and Fernando [10], poor water quality was one of the main factors related to bacterial diversity, including the emergence of *Vibrio* in the pond culture system. This result indicated that assessing water quality conditions in aquaculture ponds was essential to identify environmental situations that supported vibriosis for disease management purposes. Maintaining *Vibrio* pathogenic load in the culture system below 1000 CFU/mL was very important due to proper pond bottom and microbial management requirements. It was reported that the *V. harveyi* challenge test, at a concentration of 10^7^ CFU/mL, showed that these pathogens caused 50-98.4% of Asian marine fish and *C. altivelis* mortalities, respectively [2].

Adhesion to the body or host cells is a prerequisite for the attachment of disease-causing pathogenic bacteria. Many *Vibrio* species produce virulence factors such as capsular polysaccharides, adhesives, cytotoxins, lipopolysaccharides, and flagella. The first step of bacterial pathogenesis is through attachment to the surface of the host. Furthermore, the presence of multiple flagella facilitates bacterial motility, allowing successful infection to the host. *V. alginolyticus* has implied a mucus adhesion mechanism. Extracellular polysaccharides are secreted around the cells as capsules or loose mucus, which allow vibrios to adhere to host cells and are significantly involved in immune evasion as encapsulated pathogens exhibit increased resistance to phagocytosis, as well as complement-mediated killing [42,45]. The observations using SEM were carried out on infected fish to show and confirm the appearance of protein expression within the eye and kidney of the groupers used in this research. Bundle-forming pili (BFP) were found in SEM observations indicated by arrows in Figure-3c during organ infection. BFP is involved in bacterial autoaggregation and in mediating the initial attachment of bacteria to their host cells [46] and is postulated to initiate a long-range adhesion of bacteria with the intestinal epithelium. These bacteria, such as *V. alginolyticus* and *V. harveyi*, can express these pili in bundles to promote motility, aggregation, and twitch biofilm formation. The pili protein was one of the virulence factors that played an essential role in infection, especially in the adhesion/attachment process. The pili located at the tip used to function to mediate the attachment of bacteria. Specific binding between the pilin ends and host cell carbohydrates is mediated by the pilin itself or by a particular protein structure different from that of the pilin, namely, adhesins.

The high molecular weight protein found in whole cells and crude protein (Figures-4 and 5) is part of the siderophore system. Siderophores are high affinity and low molecular weight iron-chelating molecules secreted by various bacteria to aid iron acquisition [47]. The outer membrane is a part of the bacterial cell typically found in Gram-negative bacteria and is a pathogenetic factor that usually contains three or four main proteins. One of the common catecholate secreted by *V. alginolyticus* and *V. harveyi* is enterobactin (Ent) [48]. Since Ent is a common siderophore, the innate immune system has developed a way to bind Ent, preventing bacteria from acquiring iron. Thus, bacteria have developed other siderophores, which are encoded in the accessory genome, to counteract this. The character profile of the protein is the most dominant and virulent to its host humpback grouper (*C. altivelis*).

According to de Carvalho [49], whole cells allow the production of compounds through multistep reactions, with cofactor regeneration, with high regional and stereoselectivity, under mild and environmentally friendly operational conditions. Meanwhile, crude protein still contains the whole protein. Thus, the utilization of entire cells and crude protein from each *Vibrio* spp. in this study was used to compare recommended protein. The specificity of the receptors in each humpback grouper (Table-1) was initially measured through the hemagglutinin test to determine the presence and absence of adhesive molecules that had attachment capabilities (grouper and an adhesin in grouper and *Vibrio* spp.). Agglutination is a mechanism in the host system used to prevent the entrance of pathogens. It indicated that pathogens trapped on the extracellular surface of host cells were destroyed by macrophages opsonization and phagocytosis [50]. The occurrence of antigen clotting was caused by providing fluids or serum to the microplate. Although the hemagglutination occurred at the highest dilution, the agglutination still appeared as the antibody response of fish. The hemagglutinin pili are one of the virulence factors against the host’s defenses, besides polysaccharide capsules, lipopolysaccharides, serum resistance, and siderophore. Several bacteria use pili/fimbriae activity to infect the respiratory tract epithelium and attach through hemagglutinin filaments.

No hemagglutination was found to occur in a negative control situation without antigens and antibodies. However, hemagglutinated erythrocytes only occurred in the presence of antigens. Based on the control sample, the erythrocyte cells were found not to bind together, which led to sinking to the well plate’s bottom and were observed as a red dot in the center of the well. Meanwhile, many antigens with hemagglutinin were found to cause agglutination of erythrocyte cells, resulting in the hemagglutination and formation of a lattice structure. This structure produced a red color throughout the well [51]. Nguyen *et al*. [16] stated that fishes showed higher antibody titers and positively correlated with protective mechanisms after challenge with *V. harveyi*. This indicated that a strong antibody response and significant protection were achieved by vaccination of the *V. harveyi* infection in groupers (Table-3). The proteins naturally existed on the cell surface and were accessed by antibodies. Therefore, the outer membrane protein antibodies were able to recognize and bind all *V. harveyi* cells. The bacterial recovery from internal tissues was significantly reduced when fishes were immunized with antiserum before the *V. harveyi* infection exposure. The provision of attenuated *V. harveyi* triggered an immune response of grouper, induced a higher antibody titer, exhibited a strong bactericidal effect after the challenge, and provided good protection.

*V. harveyi* can be collected in the head, kidney, spleen, brain, skin lesions, or eye lesions [52]. The previous studies [41,42,52,53] have reported that the main target organ for the isolation of the pathogen *V. harveyi* and *V. alginolyticus* was the liver and kidney because they are the primary immune organs of fish species, and this may also be since some of the virulence determinants possessed by these pathogens augment their septicemic properties with final predisposition into the toxin neutralizing vessel in the liver and central immune in the kidney. Based on the SDS-PAGE electrophoresis results on the receptors in the grouper’s brain, eyes, and kidneys indicate that protein provides an immunological response to *V. alginolyticus* antibodies as the most immunogenic antigen. Furthermore, the receptors in these three organs stated the presence of a specific protein, which was capable of recognizing antigens on *V. alginolyticus* and *V. harveyi* (Figures-8-12). Information related to this receptor protein plays a key role in the mechanism of *Vibrio* spp. infection in groupers. When inserted into a cell or organism, immunogenic proteins are proteins that will generate a function in the complex immune system. This immune system functions to protect itself in fish that have been immunized against pathogenic antigens that have entered. Immunogenicity (development of antibodies to therapeutic proteins) is an essential concern for the safety and efficacy of protein therapy because the immune response can neutralize the activity of effective protein therapies [21,54]. The protein response to the receptor organs was often found in the eye. Analysis of protein bands that appeared in receptor organ samples after being treated with different immunogenic protein infections showed confirmation that grouper organ receptors contained immunogenic proteins following previous studies on the results of HA and *V. alginolyticus* (Table-2) and *V. harveyi* (Table-3) immunogenic protein responses.

The dot blot was a highly sensitive molecular technique used to initially identify bacteria with more remarkable antigenic similarity to the pathogen studied. The color intensity of the visible color showed a strong bond between the antigen and antibody reactions (Figure-13). Quantitatively, the color analysis of the dot blot showed the average value of the antigen-antibody response; the thicker the color, the lower the average value, and vice versa [55]. The dot blot quantification results (Table-5) showed that the kidney had a protein receptor with a specificity value of 124.24 for *V. alginolyticus* infection. The eye had a protein receptor with the highest specificity value of 106.454 for *V. harveyi* infection. The kidneys obtained the average value of the highest specificity. Therefore, the eye, brain, and kidney have specific receptors for introducing antigens against *V. algnolyticus* and *V. harveyi*.

Color intensity shows its strength between antigen and antibody reactions. At the highest dilution, the thinner dot color on the NC membrane indicates a lower antibody titer (Figure-14). The dilution titer will be used in the western blot method. The appearance of a dot that is almost evenly visible may be a possibility of a crossing reaction of antibodies between bacteria belonging to Gram-negative rods and possibly of grouper antibodies in which antibodies to the protein of *Vibrio* spp. The condition of the healthy fish due to the induction of *Vibrio* spp. Immunogenic protein was capable of triggering the production of adhesion molecules and cytokines by immune cells. The highest color quantification means the highest antibody titer produced. When an antigen infects animals, they have an immune response and an antibody (protein) that recognizes and binds tightly to the specific antigen. Each antibody recognizes only one antigen.

Based on the results of Western blotting (Figure-15) on the brain, eye, and kidney organs against *V. alginolyticus* antigen as the most immunogenic in grouper, each receptor unit showed an expressed value that was indicated by the appearance of specific protein bands, on the NC membrane blot results. Huang *et al*. [17] found that the anti-Id IgG (Fab, anti-*V. have* 27 kDa antibody) provided in the peritoneal cavity of groupers increased the cell-mediated and humoral specific immune response of *V. harveyi*. Therefore, this result provided cross-protection for groupers from the challenges of heterologous and highly virulent *V. harveyi* strains. According to molecular size, macromolecular proteins are potential immunogens, whereas molecules smaller than 10 kDa have weak immunogenic properties unless combined into immunogenic carrier proteins. The chemical structure of proteins and polysaccharides has strong immunogenic properties, although small polypeptide chains, nucleic acids, and fats can be potentially immunogenic. Chemical complexity and antigens have a direct relationship; polymeric or aggregation proteins have solid immunogenic properties than soluble monomeric proteins.

## Conclusion

The earlier diagnosis used the hemagglutination test to identify adhesion to bacterial proteins (*V. alginolyticus* and *V. harveyi*) due to the attachment mechanism of the *Vibrio* to its host by using these adhesion molecules. *V. alginolyticus* and *V. harveyi* can express BFP in bacterial autoaggregation and mediate bacteria’s initial attachment to their host cells. The most virulent protein character molecular weight profile of *V. alginolyticus* pili was 47.98 kDa with 1/64 titer dilution, and *V. harveyi* was 51.16 kDa with 1/64 titer dilution. Both *V. alginolyticus* and *V. harveyi* infections led to higher levels of specific antibodies against both pathogens. The presence of protein bands in each infected organ seemed to correspond to the isolation of bacterial protein *V. alginolyticus* 47.98 kDa and immunogenic protein *V. alginolyticus* 47.21 kDa, as well as protein isolation of bacteria *V. harveyi* 51.26 kDa and *V. harveyi* immunogenic protein 51.06 kDa. The antigen-antibody reaction of receptor specificity between *Vibrio* spp. protein matrix and anti-*Vibrio* spp. polyclonal antibody using the dot blot method to determine the dilution titer with the strong response was 1/8 antigen titer dilution. The results of the specificity test for matrix protein *Vibrio* spp. Antigens in the fishes showed that the receptors in the brain, eye, and kidney organs provided a quality and quantity level of responses, at 22.63, 53.95, and 43.15 kDa, respectively. The polyclonal IgM of anti-*V. alginolyticus* provided a cross-reaction with a greater quantity compared to the antibody of *V. harveyi*. Thus, *V. alginolyticus* bacteria were more pathogenic than *V. harveyi*. The limitation of this study is that the antibody produced remains a polyclonal antibody that has the potential to be non-specific in recognizing antigens, unwanted reaction background, and the ability to discriminate against antigens at the epitope level. In the future, the molecular character of the *V. alginolyticus* and *V. harveyi* antigens and the specific receptor organ proteins in the humpback grouper can be developed as the basis for the construction of molecular peptide-based vaccine material.

## Authors’ Contributions

UY, LW, HN, NSJ, SS, and NRC: Conception and study design. UY, LW, and HN: Caesar and acquisition of data. UY, NSJ, and NRC: Data analysis and interpretation. UY, NRC, NSJ, and HN: Drafting the manuscript. UY, NSJ, SS, and NRC: Revising the manuscript critically for important intellectual content. All authors read and approved the final manuscript.
